# Plasticity and Redundancy in Proteins Important for *Toxoplasma* Invasion

**DOI:** 10.1371/journal.ppat.1005069

**Published:** 2015-08-13

**Authors:** Karine Frénal, Dominique Soldati-Favre

**Affiliations:** Department of Microbiology and Molecular Medicine, Centre Médical Universitaire, University of Geneva, Geneva, Switzerland; University of Wisconsin Medical School, UNITED STATES

## Phenotypic Plasticity to Preserve Essential Functions

Phenotypic plasticity encompasses the versatile abilities of an organism to modify its phenotype according to changes in the environment. Such adaptations, in response to a decrease of fitness, are commonly exploited by microorganisms and plants that cannot easily escape their environment and, hence, are at risk of extinction. Functional redundancy and plasticity is therefore anticipated for obligate intracellular parasites, including members of the phylum Apicomplexa, to ensure successful host cell invasion—a critical step for their survival.

The phylum Apicomplexa includes many medically and veterinary-relevant parasites, most notably the human pathogens *Plasmodium* and *Toxoplasma gondii*, responsible for malaria and toxoplasmosis, respectively. These parasites are the most studied of the phylum, not only because of their prevalence but also because of their accessibility to genetic manipulation [1,2], which has allowed the investigation of several aspects of their lytic cycle, particularly their mechanisms of entry into the target cell.

Invasion by most Apicomplexa is a multistep process consisting of recognition, attachment, and active penetration into host cells, which has been dissected in depth in *Toxoplasma* tachyzoites [3]. Parasite propulsion into the host cell is powered by a myosin motor complex termed the glideosome. During motility and invasion, *T*. *gondii* myosin A (TgMyoA) presumably translocates adhesins at the moving junction (MJ) from the apical to the posterior pole of the parasite [4]. The MJ forms a constriction around the parasite at the point of entry into the host cell that appears as a ring structure. Apical membrane antigen 1 (AMA1) in association with a complex of rhoptry neck proteins (RONs) that are anchored into the host cortical cytoskeleton [5] are the currently known components of this MJ.

Insults imposed upon the parasite by experimental genome editing strategies, such as ablation of *TgMyoA* and *TgAMA1* genes, significantly compromised the fitness of the parasites and have now been demonstrated to elicit phenotypic plasticity. Such plasticity has uncovered informative redundancy and adaptation mechanisms. Hereafter, we compare and contrast the methodologies available to investigate the function of crucial genes in *T*. *gondii* and discuss how some experimental approaches contribute to or even favor the emergence of alternative mechanisms to ensure completion of the vital step of host cell invasion.

## A Plethora of Tools to Tackle the Function of Critical Genes in *Toxoplasma gondii*

The amenability of *T*. *gondii* to genetic manipulation has led to the development of various experimentally regulated expression systems to investigate the function of genes critical for parasite survival and, notably, for invasion [2].

Recently, the generation of gene knockouts by homologous recombination was greatly facilitated by the elimination of random integration events in parasites lacking Ku80, a protein involved in the non-homologous end-joining pathway of DNA repair [6,7]. Furthermore, Cre recombinase, either expressed upon transient transfection [8] or stably as a dimerizable Cre (DiCre) activated by rapamycin [9], efficiently excises genes flanked by LoxP sites. These refined technologies allow the generation of null mutants; however, the clonal isolation procedure and subsequent propagation of severely impaired mutants is prone to selection of spurious adaptation events resulting in an attenuation of the phenotype severity. In contrast, strategies aimed at an acute conditional depletion of a protein in a clonal population over a relatively short period of time prior to phenotypic assessment minimizes the risks of emergence of compensatory mechanisms. This holds true for the tetracycline (tet)-inducible transactivator system [10], the FKBP-destabilization domain [11], and the conditional DiCre-mediated excision system [9]. The drawback or limit of these approaches is the quasi-impossibility of fully eliminating a protein even if it falls below detectable levels, and in consequence, should never be considered or interpreted as a null mutant.

TgMyoA and TgAMA1 were initially investigated using the regulatable tet-inducible system [10,12] and shown to be critical for invasion (Table 1). More recently, null mutants for *TgMyoA* and *TgAMA1* [9,13] were isolated despite their severe phenotypes. While these genes can now be qualified as dispensable for parasite survival in vitro, subsequent studies revealed a considerable level of plasticity towards compensatory mechanisms that ensured invasion by these mutants [14–16]. These two examples are detailed below and underscore the complexity in interpreting data and the need to carefully take into account the capacity of parasite adaptation. In turn, these data reveal critical information about the function of other related genes, shedding light on their potential for functional redundancy.

**Table 1:** Summary of the phenotype and adaptation observed in the cell lines discussed in this review according to the technology used to investigate the function of the corresponding gene.

Cell line	System	Phenotype	Ref.
MyoA-cKD	Conditional transcriptional repression	16% invasion	[10]
MyoA-KO	Gene excision	25% invasion	[9,14]
MyoC-KO	Gene deletion	100% invasion	[14,15]
MyoA-cKO/MyoC-KO	Conditional gene excision/gene deletion	5% invasion	[14]
MyoA-KO/MyoC-cKD-ATc	Gene disruption/Conditional transcriptional repression	10% invasion, Peripheral relocalization of MyoC	[15]
MyoA-KO/MyoC-cKD +ATc	Gene disruption/Conditional transcriptional repression	2% invasion	[15]
GAP45-cKD	Conditional transcriptional repression	20% invasion	[19]
GAP45-cKO	Conditional gene excision	6% invasion	[14]
GAP80-KO	Gene deletion	100% invasion, Compensation by GAP45	[15]
AMA1-cKD	Conditional transcriptional repression	15% invasion	[12]
AMA1-KO	Gene excision	20% invasion, Up-regulation of AMA2	[13]
AMA1-KO	Gene disruption	10% invasion, Up-regulation of AMA2 and AMA4/RON2_L1_	[16]
AMA2-KO	Gene deletion	75% invasion	[16]
AMA1-KO/AMA2-KO	Gene disruption/gene deletion	5% invasion, Up-regulation of AMA4/RON2_L1_	[16]
ACS-KO	Gene deletion	Up-regulation of ACL	[24]
MORN1-KO	Gene excision	Basal complex formation defect, Cytokinesis defect, 24% apicoplast segregation defect	[8]
MORN1-cKD	Conditional transcriptional repression	Basal complex formation defect, Cytokinesis defect, 100% apicoplast segregation defect	[26]

## TgMyoA-KO and Recruitment of Another Myosin

**Fig 1:**
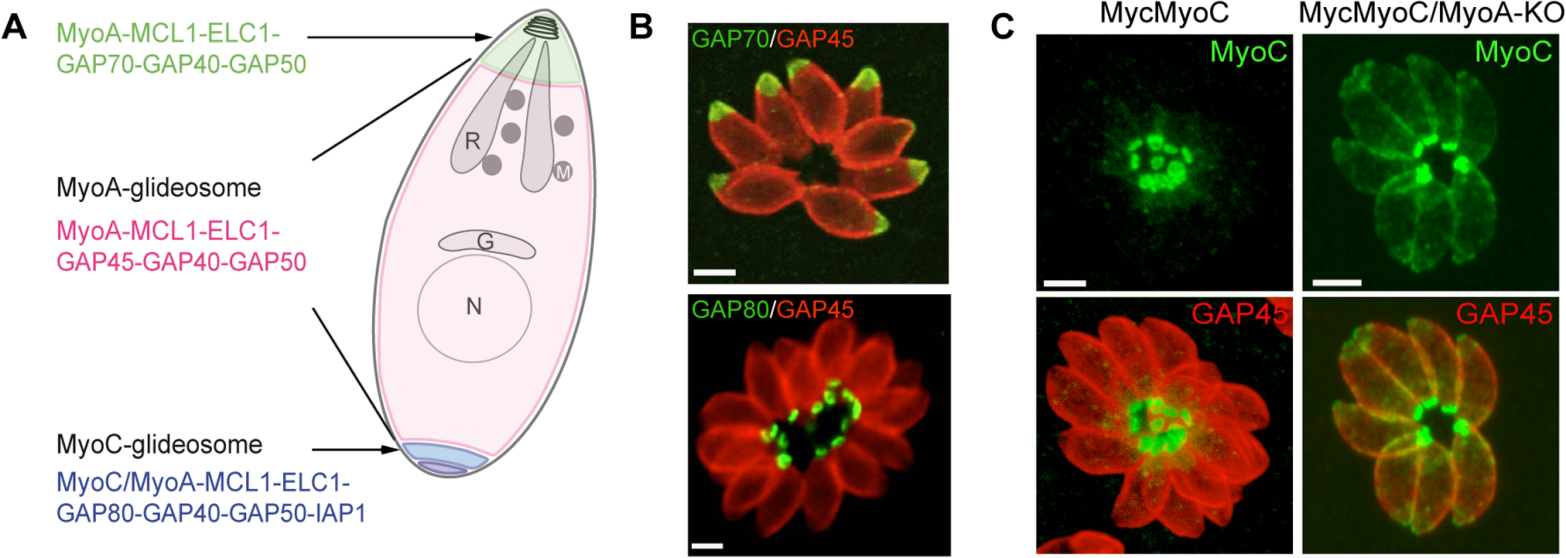
A. Schematic representation of a *T*. *gondii* tachyzoite highlighting the localization and composition of the three glideosomes. B. Localization of three glideosomes by immunofluorescence using the endogenously tagged TgGAP70 and TgGAP80 and the anti-TgGAP45 antibodies. Scale bars: 2 μm. C. Relocalization of MycMyoC to the periphery of the tachyzoite in addition to its basal localization in absence of TgMyoA in the MycMyoC-cKD/MyoA-KO strain. Scale bars: 2 μm.

The MyoA-glideosome refers to the machinery anchored to the parasite pellicle and acting as a component of gliding motility that critically contributes to invasion and egress (Fig 1A) [10]. Ablation of the *TgMyoA* gene (TgMyoA-KO) [9] raised legitimate questions regarding the validity of the gliding motility model for invasion and, alternatively, suggested the possibility of functional redundancy given the existence of 11 myosins in the *T*. *gondii* genome, with six of them belonging to the same class XIV as TgMyoA [17]. Pertinently, attempts to simultaneously disrupt *TgMyoA* and its close homologue *TgMyoC* failed [14]. Indeed, it has not been possible to isolate TgMyoA-KO clones in which *TgMyoC* was already deleted (TgMyoA-cKO/TgMyoC-KO). Moreover, conditional depletion of TgMyoC in TgMyoA-KO/TgMyoC-cKD caused a further decrease in invasion, as well, clearly indicating that TgMyoC accounts for the residual invasion seen in the TgMyoA-KO (Table 1) [14,15].

Further characterization of TgMyoC revealed that this motor belongs to the MyoC-glideosome, a complex localized to the basal polar ring, that shares an overall similar architecture with the MyoA-glideosome as well as the common myosin light chains MLC1 and ECL1 (Fig 1A and 1B) [15,18]. In parasites lacking TgMyoA, TgMyoC strikingly relocalizes along the parasite pellicle where TgMyoA normally resides (Fig 1C) [15]. This is achieved via interaction with GAP45, which typically anchors TgMyoA to the pellicle but which functionally replaces GAP80 in the recruitment of TgMyoC to the pellicle. Further evidence for plasticity was also observed in parasites lacking TgGAP80, in which TgGAP45 brings TgMyoC to the posterior pole [15,19].

Relocalization of TgMyoC immediately ensures survival of TgMyoA-KO parasites, and continuous passaging of these parasites in culture reveals an improved fitness with time and partial restoration of the invasion process.

## TgAMA1-KO and the Up-Regulation of Other AMA Family Members

Conditional knockdown of AMA1 (TgAMA1-cKD) caused a strong impairment in invasion resulting from the inability to form an intimate interaction between the parasite and the host cell at the MJ (Table 1) [12,20]. In contrast, and rather unexpectedly, TgAMA1-KO parasites were still able to enter host cells at the same speed as wild-type parasites through the establishment of a visible MJ [13]. Intriguingly, the invasion efficiency of the TgAMA1-KO was superior to that of the TgAMA1-cKD, suggesting that a compensatory mechanism was selected during the continuous passaging of the mutant [13,16]. With the view that the MJ serves as a platform for the transmission of the force generated by the glideosome [21], TgAMA1 had to be replaced by a functionally redundant protein capable of bridging the RONs and the parasite actomyosin system. In this context, two homologues of TgAMA1 were readily identified: TgAMA2 and the sporozoite AMA1 (TgAMA3), which specifically interacts with the sporozoite RON2 (TgRON2_L2_) [22]. Both proteins represent plausible, functionally redundant candidates. Concordantly, in the TgAMA1-KO, TgAMA2 expression was shown to be up-regulated at the transcript level as monitored by qPCR [13,16] and was detectable at the MJ interacting with TgRON2 in place of TgAMA1 [16]. In addition, continuous in vitro culture over 12 months showed an increasing invasion rate and, therefore, an improved fitness. Interestingly, in the TgAMA1-KO/TgAMA2-KO double mutant, TgAMA4, which also shares sequence similarities with TgAMA1 and TgAMA3, was found to be overexpressed. This overexpression was accompanied by the concomitant up-regulation of the specific TgAMA4 ligand TgRON2_L1_ [16].

This series of AMA-RON2 pairs that can be mobilized upon successive gene deletions highlights the considerable degree of parasite plasticity and the critical role of the MJ for host cell entry.

## Plasticity Operates in Other Apicomplexa and in Processes Outside of Invasion

A striking and comparable example of genome plasticity related to the invasion process has been reported for the etiologic agent of human malaria, *Plasmodium falciparum*. Indeed, upon gene deletion, this parasite is able to redeploy a machinery composed of adhesin ligands organized hierarchically to ensure invasion via alternative pathways [23].

Carbon metabolism generates metabolites critically needed for biomass production and cell survival. Upon genetic intervention, the parasites are also expected to adjust and compensate for the deletion of genes implicated in any of these vital processes. For example, the pool of cytoplasmic acetyl-CoA is an essential metabolite needed for acetylation of proteins and lipogenesis and can be produced from citrate through the action of ATP citrate lyase (ACL) or from acetate by acetyl-CoA synthetase (ACS). In *Toxoplasma* tachyzoites, TgACS and TgACL can be individually deleted without noticeable phenotypes but cannot be deleted in combination. While TgACL is barely detectable in wild-type parasites, it is readily up-regulated in TgACS-KO parasites to adjust the level of acetyl-CoA required for proper growth [24]. This response to loss of fitness illustrates a rapid adaptation to unfavorable conditions by up-regulation of a complementary metabolic pathway.

Lastly, organelle biogenesis and cell division are fundamental events that can also challenge the faculty of tachyzoite adaptation. Such an adaptation was reported upon the disruption of the membrane occupation and recognition nexus protein 1 (TgMORN1), a protein that associates with several structures implicated in cell division [25]. While conditional depletion of TgMORN1 (TgMORN1-cKD) showed impairment in basal complex formation, cytokinesis, and apicoplast (plastid-like organelle) inheritance [26], a TgMORN1-KO mutant, isolated following Cre recombinase excision, presented an attenuation of the phenotype severity, in particular for apicoplast segregation [8]. The apicoplast hosts essential metabolic pathways [27] and is rapidly lost upon multiple rounds of division in the TgMORN1-cKD, indicating that MORN1 is critical for the survival of the parasite. It is therefore surprising that TgMORN1-KO parasites can be propagated in culture with only a quarter of them without apicoplast. These results suggest an adaptation phenomenon possibly because of a functional redundancy since other MORN-containing proteins are present in the *T*. *gondii* genome.

## Concluding Remarks

*T*. *gondii* is an experimentally versatile member of the Apicomplexan phylum that needs to be taken seriously when it comes to the emergence and study of compensatory mechanisms. The few examples discussed here only show the tip of the iceberg. The frequency at which the compensatory changes occur has not yet been determined, and thus the mechanisms responsible have not yet been unraveled; however, they might include epigenetic processes such as chromatin remodeling or changes in protein conformation and stability.

The gene knockout technologies available for *Toxoplasma* [2] and *Plasmodium* [1] are further potentiated by the CRISPR/Cas9-based genome editing strategy [28–32]. This genome editing engenders off-target effects, and thus it becomes even more crucial to be aware of the strengths and weaknesses of these approaches. The acute, conditional depletion of a protein remains the panacea to study its function. As illustrated above, the complete deletion of a gene opens a new dimension of investigations that deserve to be globally scrutinized prior to drawing conclusions. We are entering an area where global phenotypic analyses become feasible and should be used to embrace the phenotypic complexity of a single gene deletion and, incidentally, to avoid pitfalls.
